# Supplementary material for: Influence of Lactitol and Psyllium on Bowel Function in Constipated Indian Volunteers: A Randomized, Controlled Trial
Source: Nutrients. 2019 May 21;11(5):1130. doi: 10.3390/nu11051130 (PMC6566185; doi:10.3390/nu11051130)
Supplement: Supplementary file 1 [file nutrients-11-01130-s001.pdf]

Supplemental Figures and Tables

Influence of Lactitol and Psyllium on Bowel Function in Constipated Indian Volunteers: A Randomized, Controlled Trial

Jing Cheng [1], Julia Tennilä [1], Lotta Stenman [1], Alvin Ibarra [1,*], Mandhir Kumar [2], Kamlesh Kumar Gupta [3], Shyam Sundar Sharma [4], Dhiman Sen [5], Sandeep Garg [6], Mukund Penurkar [7], Santosh Kumar [8] and Arthur C. Ouwehand [1].

[1] DuPont, Global Health & Nutrition Science, Danisco Sweeteners Oy, Sokeritehtaantie 20, FI-02460 Kantvik, Finland; jing.cheng@dupont.com (J.C.); julia.tennila@outlook.com (J.T.); lotta.stenman3@gmail.com (L.S.); arthur.ouwehand@dupont.com (A.O.)

[2] Department of Nephrology, Sir Ganga Ram Hospital, Old Rajindra Nagar. New Delhi 110060, India; mandhirkr@yahoo.co.in (M.K.)

[3] Department of Medicine, King George's Medical University Chowk, Lucknow 226003, Uttar Pradesh, India; kamleshkgmu@rediffmail.com (K.K.G.)

[4] Department of Gastroenterology, Sawai Man Singh Hospital. JLN Marg, Jaipur 302004, Rajasthan, India; shyamsharma4@rediffmail.com (S.S.S.)

[5] Department of Clinical Trial & Research, Apollo Gleneagles Hospitals, Kolkata 58, Canal Circular Road, Kolkata 700054, West Bengal, India; dhimansen2004@yahoo.com (D.S.)

[6] Department of Medicine, Maulana Azad Medical College & Associated Lok Nayak Hospital, Bahadur shah Zafar Marg, New Delhi 110002, India; drsandeepgargmamc@gmail.com (S.G.)

[7] Department of Medicine, Sanjeevan Hospital, Plot No 23, Off. Karve Road, Erandwane, Pune 411 004, India; drpenurkarm@gmail.com (M.P.)

[8] Department of SAS & Biostatistics – JSS Medical Research, JSS Medical Research India Private Limited, Mathura Road Sector 27 D, Faridabad, Haryana 121003, India; santosh1.kumar@jssresearch.com (S.K.)

* Correspondence: alvin.ibarra@dupont.com; Tel.: +358-40-1678-509

SUPPLEMENTAL FIGURES AND FIGURE LEGENDS

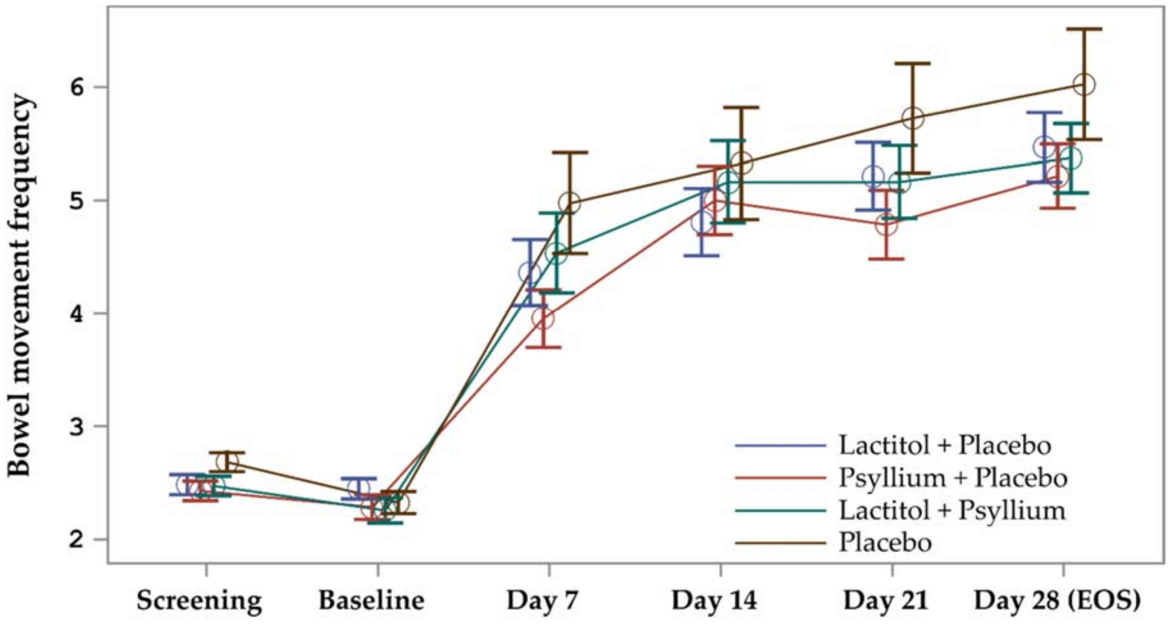

Figure S1. Bowel movement frequency by visit-ITT population (N=172) over time. The number of bowel movement frequency is presented as mean and standard error of mean for each treatment arm. EOS - End of Study; ITT - intention-to-treat.

SUPPLEMENTAL TABLES

Table S1. List of Investigators and Clinics

Site	Investigator's Name	Site Name and Address
01	Dr. Mandhir Kumar	Sir Ganga Ram Hospital, Department of Nephrology, Old Rajindra Nagar, New Delhi-110060, India
02	Dr. Kamlesh Kumar Gupta	King George's Medical University Chowk, Lucknow, Uttar Pradesh- 226003, India
03	Dr. Shyam Sundar Sharma	Medical College and Hospital, JLN Marg Jaipur-302001, Rajasthan, India
04	Dr. Dhiman Sen	Apollo Gleneagles Hospitals, Kolkata 58, Canal Circular Road, Kolkata-700054 West Bengal, India
05	Dr. Sandeep Garg	Maulana Azad Medical College & Associated Lok Nayak Hospital, Bahadur shah Jafar Marg, New Delhi-110002, India
06	Dr. Mukund Penurkar	Sanjeevan Hospital, Plot No 23, Off. Karve Road, Erandwane, Pune 411 004, India

Table S2. Formulation of IPs.

Ingredients (g)		*Arm 1* *Lactitol*	*Arm 2* *Psyllium*	*Arm 3* *Psyllium+Lactitol*	*Arm 4* *Placebo*
Active Ingredients	**Psyllium**	-	3.5	3.5	-
	**Lactitol**	10	-	10	-
Excipients	**Sucrose**	21	24	21	24
	**Instant waxy Maize starch**	3.75	-	-	3.75
	**Citric acid**	0.55	0.55	0.55	0.55
	**Trisodium citrate**	0.15	0.15	0.15	0.15
	**Carotene powder**	0.10	0.10	0.10	0.10
	**CF- Orange flavor**	0.25	0.25	0.25	0.25
Total g per sachet		35.8	28.5	35.55	28.8

Table S3. Summary of Relevant Medical History and Surgical History - ITT Population (N=172)

Body system/Category, n[†] (%)*	Treatment Group				
	Arm 1 (Lactitol, N=47)	Arm 2 (Psyllium, N=42)	Arm 3 (Lactitol+Psyllium, N=43)	Arm 4 (Placebo, N=40)	Overall (N=172)
**Clinically relevant medical history**					
Yes	14 (29.8)	8 (19.0)	6 (14.0)	11 (27.5)	39 (22.7)
No	33 (70.2)	34 (81.0)	37 (86.0)	29 (72.5)	133 (77.3)
P-Value [#]	0.8142	0.3645	0.1265		
If Yes [1]					
**Endocrine disorders**	4 (28.6)	1 (12.5)	2 (33.3)	2 (18.2)	9 (23.1)
Current status [2]					
Past	-	-	-	-	-
Ongoing	4 (100.0)	1 (100.0)	2 (100.0)	2 (100.0)	9 (100.0)
On treatment currently					
Yes	4 (100.0)	1 (100.0)	2 (100.0)	2 (100.0)	9 (100.0)
No	-	-	-	-	-
**Gastrointestinal disorders**	3 (21.4)	-	2 (33.3)	2 (18.2)	7 (17.9)
Current status [2]					
Past	-	-	-	-	-
Ongoing	3 (100.0)	-	2 (100.0)	2 (100.0)	7 (100.0)
On treatment currently					
Yes	3 (100.0)	-	2 (100.0)	-	5 (71.4)
No	-	-	-	2 (100.0)	2 (28.6)
**General disorders and administration**	-	1 (12.5)	1 (16.7)	2 (18.2)	4 (10.3)
Current status [2]					
Past	-	-	-	-	-
Ongoing	-	1 (100.0)	1 (100.0)	2 (100.0)	4 (100.0)
On treatment currently					
Yes	-	1 (100.0)	1 (100.0)	2 (100.0)	4 (100.0)
No	-	-	-	-	-
**Infections and infestations**	-	-	-	1 (9.1)	1 (2.6)
Current status [2]					
Past	-	-	-	-	-
Ongoing	-	-	-	1 (100.0)	1 (100.0)
On treatment currently					
Yes	-	-	-	1 (100.0)	1 (100.0)
No	-	-	-	-	-
**Investigations**	-	1 (12.5)	-	-	1 (2.6)
Current status [2]					
Past	-	1 (100.0)	-	-	1 (100.0)

	Treatment Group				
Body system/Category, n† (%)*	Arm 1 (Lactitol, N=47)	Arm 2 (Psyllium, N=42)	Arm 3 (Lactitol+Psyllium, N=43)	Arm 4 (Placebo, N=40)	Overall (N=172)
Ongoing	-	-	-	-	-
On treatment currently					
Yes	-	-	-	-	-
No	-	1 (100.0)	-	-	1 (100.0)
**Metabolism and nutrition disorders**	4 (28.6)	3 (37.5)	2 (33.3)	4 (36.4)	13 (33.3)
Current status [2]					
Past	-	-	-	-	-
Ongoing	4 (100.0)	3 (100.0)	2 (100.0)	4 (100.0)	13 (100.0)
On treatment currently					
Yes	4 (100.0)	3 (100.0)	2 (100.0)	4 (100.0)	13 (100.0)
No	-	-	-	-	-
**Nervous system disorders**	-	-	-	1 (9.1)	1 (2.6)
Current status [2]					
Past	-	-	-	-	-
Ongoing	-	-	-	1 (100.0)	1 (100.0)
On treatment currently					
Yes	-	-	-	1 (100.0)	1 (100.0)
No	-	-	-	-	-
**Reproductive system and breast disorders**	-	-	-	1 (9.1)	1 (2.6)
Current status [2]					
Past	-	-	-	1 (100.0)	1 (100.0)
Ongoing	-	-	-	-	-
On treatment currently					
Yes	-	-	-	-	-
No	-	-	-	1 (100.0)	1 (100.0)
**Respiratory, thoracic, and mediastinal**	-	1 (12.5)	-	-	1 (2.6)
Current status [2]					
Past	-	-	-	-	-
Ongoing	-	1 (100.0)	-	-	1 (100.0)
On treatment currently					
Yes	-	1 (100.0)	-	-	1 (100.0)
No	-	-	-	-	-
**Surgical and medical procedures**	4 (28.6)	-	1 (16.7)	1 (9.1)	6 (15.4)
Current status [2]					
Past	4 (100.0)	-	1 (100.0)	1 (100.0)	6 (100.0)
Ongoing	-	-	-	-	-
On treatment currently					
Yes	-	-	-	-	-
No	4 (100.0)	-	1 (100.0)	1 (100.0)	6 (100.0)

Body system/Category, n† (%)*	Treatment Group<br>Arm 1 (Lactitol, N=47)	Arm 2 (Psyllium, N=42)	Arm 3 (Lactitol+Psyllium, N=43)	Arm 4 (Placebo, N=40)	Overall (N=172)
**Vascular disorders**	3 (21.4)	3 (37.5)	3 (50.0)	5 (45.5)	14 (35.9)
Current status [2]					
Past	-	-	-	-	-
Ongoing	3 (100.0)	3 (100.0)	3 (100.0)	5 (100.0)	14 (100.0)
On treatment currently					
Yes	3 (100.0)	3 (100.0)	3 (100.0)	5 (100.0)	14 (100.0)
No	-	-	-	-	-

Note:

Zero frequencies are denoted as -.

† Number of subjects.

*Respective column header group counts were used as denominators for percentage calculations.

-- [1] Percentage was calculated using 'Yes' count as denominator.

-- [2] Percentage for 'current status' and 'on treatment currently' was calculated taking SOC count as denominator.

P-value was calculated using chi-square/Fisher's exact test for comparison between Active (Lactitol 10 g + Placebo, Psyllium 3.5 g + Placebo and Lactitol 10 g + Psyllium 3.5 g) vs Placebo group.

Abbreviations: intention-to-treat (ITT), system organ class (SOC).

Table S4. Summary of Concomitant Medications- Safety Population (N=172)

Category, n (%)/Therapeutic class [1]/ Generic Name [2]	Treatment Group				
	**Arm 1 (Lactitol, N=47)**	**Arm 2 (Psyllium, N=42)**	**Arm 3 (Lactitol+Psyllium, N=43)**	**Arm 4 (Placebo, N=40)**	**Overall (N=172)**
**Analgesics**	1 (2.1)	0	1 (2.3)	0	2 (1.2)
**Paracetamol**	1 (100.0)	0	1 (100.0)	0	2 (100.0)
Anti-inflammatory and antirheumatic products	0	0	1 (2.3)	0	1 (0.6)
Nimesulide	0	0	1 (100.0)	0	1 (100.0)
**Drugs for acid-related disorders**	0	0	2 (4.7)	1 (2.5)	3 (1.7)
Esomeprazole sodium	0	0	1 (50.0)	0	1 (33.3)
Omeprazole	0	0	0	1 (100.0)	1 (33.3)
Pantoprazole sodium sesquihydrate	0	0	2 (100.0)	0	2 (66.7)
**Drugs for constipation**	0	0	1 (2.3)	0	1 (0.6)
Bisacodyl	0	0	1 (100.0)	0	1 (100.0)
**Drugs for functional gastrointestinal disorders**	0	0	2 (4.7)	0	2 (1.2)
Drotaverine hydrochloride	0	0	2 (100.0)	0	2 (100.0)
**Mineral supplements**	0	0	1 (2.3)	1 (2.5)	2 (1.2)
Calcium	0	0	1 (100.0)	0	1 (50.0)
Calcium carbonate, Cholecalciferol	0	0	0	1 (100.0)	1 (50.0)
**Vitamins**	0	3 (7.1)	1 (2.3)	0	4 (2.3)
Cholecalciferol	0	0	1 (100.0)	0	1 (25.0)
Minerals numbers, Vitamins numbers	0	2 (66.7)	0	0	2 (50.0)
Vitamin B numbers	0	1 (33.3)	0	0	1 (25.0)

Category, n (%)/Therapeutic class [1]/ Generic Name [2]	Treatment Group				
	Arm 1 (Lactitol, N=47)	Arm 2 (Psyllium, N=42)	Arm 3 (Lactitol+Psyllium, N=43)	Arm 4 (Placebo, N=40)	Overall (N=172)

Note:

N: number of subjects.

[1] Percentage of therapeutic class was calculated by taking the count of the corresponding column header group as denominator.

[2] Percentage of generic name of drug was calculated from the respective drug class.

Medications were coded using the WHO Drug Dictionary version of March 1, 2016 or later. Patients may have taken more than one medication in a drug class or preferred name. Patients are only counted once for each drug class or preferred name summary.

Table S5. Summary of Actual value and Change from Baseline in Evaluation of food intake by 24 hours - ITT population (N=172)

Statistics	Treatment Group							
	Actual value				Change from Baseline			
	Arm 1 (Lactitol, N=47)	Arm 2 (Psyllium, N=42)	Arm 3 (Lactitol+ Psyllium, N=43)	Arm 4 (Placebo, N=40)	Arm 1 (Lactitol, N=47)	Arm 2 (Psyllium, N=42)	Arm 3 (Lactitol+ Psyllium, N=43)	Arm 4 (Placebo, N=40)
<u>**Total Calories**</u>								
Baseline								
n	47	42	43	40	-	-	-	-
Mean	1270.8	1253.0	1223.9	1176.4	-	-	-	-
SD	449.54	405.01	408.64	434.69	-	-	-	-
Median	1193.0	1262.0	1140.0	1091.5	-	-	-	-
Q1	1038.0	991.0	907.0	832.0	-	-	-	-
Q3	1326.0	1538.0	1513.0	1482.5	-	-	-	-
Range (Min.-Max.)	(704-3342)	(189-2203)	(601-2045)	(500-2173)	-	-	-	-
Missing	0	0	0	0	-	-	-	-
Day 28 (EOS)								
n	47	42	43	40	47	42	43	40
Mean	1270.7	1217.1	1292.7	1266.9	-0.1	-36.0	68.8	90.5
SD	398.96	347.74	369.81	422.56	489.09	444.45	450.14	343.59
Median	1319.0	1214.0	1303.0	1207.5	64.0	-128.5	64.0	38.0
Q1	907.0	946.0	1002.0	963.0	-205.0	-323.0	-210.0	-108.0
Q3	1599.0	1460.0	1530.0	1497.0	294.0	130.0	425.0	380.5
Range (Min.-Max.)	(360-2142)	(199-1867)	(640-1983)	(601-2214)	(-1727-969)	(-743-1678)	(-1270-807)	(-573-799)
Missing	0	0	0	0	0	0	0	0
P-value [1]	0.9989	0.6027	0.3218	0.1038	-	-	-	-
LS Mean Estimate	1256.341	1209.305	1295.892	1288.443	23.5737	-23.4629	63.1242	55.6760
Difference Estimate [2]	-	-	-	-	-32.102	-79.139	7.448	
SE [3]	-	-	-	-	75.942	77.902	77.369	
95% CI (L. – U.)	-	-	-	-	(-211.770 - 147.5651)	(-263.442 - 105.1640)	(-175.594 - 190.4903)	
P-value [4,5]	-	-	-	-	0.9506	0.6080	0.9993	
<u>**Carbohydrate (g)**</u>								
Baseline								
n	47	42	43	40	-	-	-	-
Mean	185.19	180.98	183.11	173.85	-	-	-	-
SD	72.899	60.321	61.962	67.065	-	-	-	-
Median	170.70	182.90	170.30	167.35	-	-	-	-
Q1	151.10	135.10	136.70	122.95	-	-	-	-
Q3	207.30	224.60	218.50	201.55	-	-	-	-

Statistics	Treatment Group							
	Actual value				Change from Baseline			
	Arm 1 (Lactitol, N=47)	Arm 2 (Psyllium, N=42)	Arm 3 (Lactitol+ Psyllium, N=43)	Arm 4 (Placebo, N=40)	Arm 1 (Lactitol, N=47)	Arm 2 (Psyllium, N=42)	Arm 3 (Lactitol+ Psyllium, N=43)	Arm 4 (Placebo, N=40)
Range (Min.-Max.)	(72.3-469.5)	(33.1-331.0)	(57.4-321.8)	(78.5-351.7)	-	-	-	-
Missing	0	0	0	0	-	-	-	-
Day 28 (EOS)								
n	47	42	43	40	47	42	43	40
Mean	182.65	177.94	184.60	181.43	-2.53	-3.04	1.49	7.58
SD	63.535	48.691	60.620	62.703	77.273	68.403	69.366	55.726
Median	181.50	170.10	184.40	172.40	5.00	-6.95	4.20	6.75
Q1	131.30	140.60	136.80	140.10	-41.60	-41.60	-41.40	-26.10
Q3	230.30	204.80	231.60	212.45	54.60	29.40	49.20	44.20
Range (Min.-Max.)	(50.2-341.6)	(101.5-312.1)	(83.7-298.6)	(66.1-346.3)	(-279.8-114.4)	(-162.0-221.8)	(-184.4-123.0)	(-130.9-146.4)
Missing	0	0	0	0	0	0	0	0
P-value [1]	0.8231	0.7746	0.8890	0.3952	-	-	-	-
LS Mean Estimate	181.3582	177.9448	183.6337	183.9733	0.3542	-3.0593	2.6296	2.9693
Difference Estimate [2]	-	-	-	-	-2.615	-6.029	-0.340	
SE [3]	-	-	-	-	11.584	11.882	11.821	
95% CI (L. – U.)	-	-	-	-	(-30.0228 - 24.7926)	(-34.1414 - 22.0843)	(-28.3071 - 27.6277)	
P-value [4,5]	-	-	-	-	0.9917	0.9195	1.0000	
<u>**Fat (g)**</u>								
Baseline								
n	47	42	43	40	-	-	-	-
Mean	40.375	40.650	37.279	36.440	-	-	-	-
SD	17.7835	18.4447	16.6715	16.2787	-	-	-	-
Median	37.770	38.115	32.740	34.265	-	-	-	-
Q1	29.540	28.700	27.610	24.300	-	-	-	-
Q3	47.500	51.640	47.190	43.115	-	-	-	-
Range (Min.-Max.)	(13.64-114.08)	(2.58-106.17)	(8.31-80.20)	(9.66-76.26)	-	-	-	-
Missing	0	0	0	0	-	-	-	-
Day 28 (EOS)								
n	47	42	43	40	47	42	43	40
Mean	41.356	40.477	42.214	41.710	0.981	-0.173	4.935	5.270
SD	17.4473	13.2147	14.6352	18.5824	21.1536	19.9522	19.7353	16.1524
Median	39.280	37.405	42.930	38.130	0.300	-2.935	4.270	3.895
Q1	27.790	29.040	30.080	25.705	-6.810	-12.510	-6.420	-3.655
Q3	51.620	48.290	51.170	53.600	8.320	7.570	20.260	15.895

Statistics	Treatment Group							
	Actual value				Change from Baseline			
	**Arm 1 (Lactitol, N=47)**	**Arm 2 (Psyllium, N=42)**	**Arm 3 (Lactitol+ Psyllium, N=43)**	**Arm 4 (Placebo, N=40)**	**Arm 1 (Lactitol, N=47)**	**Arm 2 (Psyllium, N=42)**	**Arm 3 (Lactitol+ Psyllium, N=43)**	**Arm 4 (Placebo, N=40)**
Range (Min.-Max.)	(7.04-95.74)	(21.78-76.56)	(18.64-82.06)	(17.53-92.91)	(-57.96-64.72)	(-48.86-54.92)	(-43.04-43.26)	(-27.99-41.41)
Missing	0	0	0	0	0	0	0	0
P-value [1]	0.7521	0.9555	0.1086	**0.0458**	-	-	-	-
LS Mean Estimate	40.8604	39.9339	42.6697	42.3720	2.1073	1.1808	3.9167	3.6189
Difference Estimate [2]	-	-	-	-	-1.512	-2.438	0.298	
SE [3]	-	-	-	-	3.292	3.382	3.351	
95% CI (L. – U.)	-	-	-	-	(-9.3002 - 6.2770)	(-10.4402 - 5.5640)	(-7.6308 - 8.2263)	
P-value [4,5]	-	-	-	-	0.9383	0.8078	0.9995	
**Protein (g)**								
Baseline								
n	47	42	43	40	-	-	-	-
Mean	41.983	40.979	39.289	38.406	-	-	-	-
SD	16.0282	18.1851	14.2051	20.3680	-	-	-	-
Median	39.320	37.935	38.490	35.010	-	-	-	-
Q1	31.690	29.370	28.260	24.955	-	-	-	-
Q3	45.810	47.300	50.270	42.140	-	-	-	-
Range (Min.-Max.)	(18.53-110.26)	(8.22-114.87)	(14.02-83.48)	(15.61-115.46)	-	-	-	-
Missing	0	0	0	0	-	-	-	-
Day 28 (EOS)								
n	47	42	43	40	47	42	43	40
Mean	42.273	42.367	43.409	40.971	0.290	1.388	4.120	2.565
SD	17.7173	17.4161	15.0613	18.0836	21.1048	16.4870	16.8589	17.5414
Median	39.170	38.445	44.140	36.055	-0.020	-1.085	2.680	2.310
Q1	29.600	31.290	32.550	32.025	-6.860	-7.350	-5.380	-5.835
Q3	50.120	46.720	51.970	46.185	8.180	10.520	11.530	10.490
Range (Min.-Max.)	(8.83-95.60)	(22.43-85.68)	(21.34-95.42)	(15.33-93.32)	(-57.27-66.92)	(-30.50-74.60)	(-36.06-62.35)	(-63.57-60.53)
Missing	0	0	0	0	0	0	0	0
P-value [1]	0.9255	0.5883	0.1166	0.3607	-	-	-	-
LS Mean Estimate	41.5114	42.0662	43.8495	41.7081	1.2788	1.8335	3.6169	1.4755
Difference Estimate [2]	-	-	-	-	-0.197	0.358	2.141	
SE [3]	-	-	-	-	3.287	3.371	3.348	
95% CI (L. – U.)	-	-	-	-	(-7.9745 - 7.5810)	(-7.6188 - 8.3349)	(-5.7807 - 10.0635)	
P-value [4,5]	-	-	-	-	0.9998	0.9991	0.8554	

Statistics	Treatment Group							
	Actual value				Change from Baseline			
	**Arm 1 (Lactitol, N=47)**	**Arm 2 (Psyllium, N=42)**	**Arm 3 (Lactitol+ Psyllium, N=43)**	**Arm 4 (Placebo, N=40)**	**Arm 1 (Lactitol, N=47)**	**Arm 2 (Psyllium, N=42)**	**Arm 3 (Lactitol+ Psyllium, N=43)**	**Arm 4 (Placebo, N=40)**
**<u>Fiber (g)</u>**								
Baseline								
n	47	42	43	40	-	-	-	-
Mean	3.86	3.68	4.23	3.55	-	-	-	-
SD	1.835	1.318	2.400	1.642	-	-	-	-
Median	3.60	3.70	3.80	3.25	-	-	-	-
Q1	2.70	2.60	2.40	2.30	-	-	-	-
Q3	4.90	4.50	5.40	4.35	-	-	-	-
Range (Min.-Max.)	(1.2-10.8)	(0.4-6.3)	(0.4-11.1)	(1.6-8.3)	-	-	-	-
Missing	0	0	0	0	-	-	-	-
Day 28 (EOS)								
n	47	42	43	40	47	42	43	40
Mean	3.34	3.63	3.63	4.00	-0.51	-0.05	-0.59	0.45
SD	1.522	1.274	1.579	2.777	2.032	1.635	1.957	1.940
Median	3.30	3.30	3.70	3.05	-0.40	-0.10	-0.20	0.40
Q1	2.40	2.80	2.40	2.55	-1.60	-1.10	-2.10	-0.90
Q3	4.40	4.60	4.50	4.30	0.70	0.70	0.80	1.05
Range (Min.-Max.)	(0.6-8.5)	(1.8-6.6)	(1.2-8.6)	(1.3-13.9)	(-8.3-2.8)	(-3.0-4.8)	(-6.3-3.4)	(-2.9-8.2)
Missing	0	0	0	0	0	0	0	0
P-value [1]	**0.0891**	0.8439	**0.0535**	0.1504	-	-	-	-
LS Mean Estimate	3.3387	3.7032	3.4452	4.1300	-0.4962	-0.1317	-0.3897	0.2951
Difference Estimate [2]	-	-	-	-	-0.791	-0.427	-0.685	
SE [3]	-	-	-	-	0.346	0.354	0.355	
95% CI (L. – U.)	-	-	-	-	(-1.6088 - 0.0262)	(-1.2651 - 0.4115)	(-1.5250 - 0.1554)	
P-value [4,5]	-	-	-	-	**0.0602**	0.4789	0.1359	
**<u>Liquid intake (mL)</u>**								
Baseline								
n	47	41	43	39	-	-	-	-
Mean	1104.3	917.1	973.3	1061.5	-	-	-	-
SD	984.21	576.37	717.50	826.97	-	-	-	-
Median	900.0	850.0	750.0	850.0	-	-	-	-
Q1	500.0	550.0	600.0	500.0	-	-	-	-
Q3	1200.0	1000.0	1000.0	1200.0	-	-	-	-
Range (Min.-Max.)	(0-4500)	(300-3100)	(300-3400)	(150-4350)	-	-	-	-

Statistics	Treatment Group							
	Actual value				Change from Baseline			
	Arm 1 (Lactitol, N=47)	Arm 2 (Psyllium, N=42)	Arm 3 (Lactitol+ Psyllium, N=43)	Arm 4 (Placebo, N=40)	Arm 1 (Lactitol, N=47)	Arm 2 (Psyllium, N=42)	Arm 3 (Lactitol+ Psyllium, N=43)	Arm 4 (Placebo, N=40)
Missing	0	1	0	1	-	-	-	-
Day 28 (EOS)								
n	47	41	42	40	47	40	42	39
Mean	862.8	959.8	977.4	921.3	-241.5	36.3	-11.9	-132.1
SD	497.98	557.59	663.89	666.50	675.29	371.76	613.35	392.95
Median	700.0	800.0	750.0	750.0	0.0	0.0	0.0	0.0
Q1	600.0	600.0	600.0	600.0	-300.0	-150.0	-250.0	-300.0
Q3	1000.0	1000.0	1050.0	1000.0	150.0	125.0	300.0	150.0
Range (Min.-Max.)	(150-3000)	(150-2700)	(150-2900)	(150-3600)	(-3000-600)	(-900-1000)	(-1400-2000)	(-1000-600)
Missing	0	1	1	0	0	2	1	1
P-value [1]	**0.0181**	0.5410	0.9005	**0.0425**	-	-	-	-
LS Mean Estimate	819.1806	1019.774	996.9801	909.6307	-205.522	-4.9288	-27.7223	-115.072
Difference Estimate [2]	-	-	-	-	-90.450	110.143	87.349	
SE [3]	-	-	-	-	88.117	91.678	90.497	
95% CI (L. – U.)	-	-	-	-	(-299.003 - 118.1027)	(-106.839 - 327.1250)	(-126.838 - 301.5362)	
P-value [4,5]	-	-	-	-	0.6011	0.4814	0.6441	

Note:

[1] *P*-value was calculated using paired t-test in each treatment group comparing assessments on consecutive visits with baseline results.

[2] Difference estimate indicates LS Mean Difference of [(Lactitol 10 g + Placebo) – Placebo], [(Psyllium 3.5 g + Placebo) – Placebo], and [(Lactitol 10 g + Psyllium 3.5 g) – Placebo].

[3] SE indicates Standard Error of LS Mean Differences of [(Lactitol 10 g + Placebo) – Placebo], [(Psyllium 3.5 g + Placebo) – Placebo], and [(Lactitol 10 g + Psyllium 3.5 g) – Placebo].

[4] *P*-value for (Lactitol 10 g + Placebo), (Psyllium 3.5 g + Placebo), and (Lactitol 10 g + Psyllium 3.5 g) group indicates significance of treatment differences with Placebo using Dunnett's test at 5% level of significance.

[5] Analysis of covariance (ANCOVA) model included treatment and population stratum (veg. /non-veg.) as factor and baseline value as covariate.

Abbreviations: intention-to-treat (ITT), end-of-study (EOS), standard deviation (SD), Standard Error (SE), Lower Limit (L), Upper Limit (U), Confidence Interval (CI).

Table S6. Summary of Adverse Events- Overall summary- Safety Population (N=172)

	Treatment Group				
**Category, n† (%)* [n]#**	**Lactitol 10 g + Placebo (N=47)**	**Psyllium 3.5 g + Placebo (N=42)**	**Lactitol 10 g + Psyllium 3.5 g (N=43)**	**Placebo (N=40)**	**Overall (N=172)**
**Total number of AEs reported**	6	1	5	2	14
**Total number of TEAEs reported**	4	0	4	0	8
**Total number of Non-TEAEs reported**	2	1	1	2	6
**Subjects reporting any AEs**	3 (6.4) [6]	1 (2.4) [1]	4 (9.3) [5]	1 (2.5) [2]	9 (5.2) [14]
Subjects reporting 1 AE	1 (2.1) [1]	1 (2.4) [1]	3 (7.0) [3]	0	5 (2.9) [5]
Subjects reporting >1 AE	2 (4.3) [5]	0	1 (2.3) [2]	1 (2.5) [2]	4 (2.3) [9]
**Subjects reporting any TEAEs**	2 (4.3) [4]	0	3 (7.0) [4]	0	5 (2.9) [8]
Subjects reporting 1 TEAE	1 (2.1) [1]	0	2 (4.7) [2]	0	3 (1.7) [3]
Subjects reporting >1 TEAE	1 (2.1) [3]	0	1 (2.3) [2]	0	2 (1.2) [5]
**Subjects reporting AEs by seriousness**					
Yes	0	0	0	0	0
No	3 (6.4) [6]	1 (2.4) [1]	4 (9.3) [5]	1 (2.5) [2]	9 (5.2) [14]
**Subjects reporting AEs with severity**					
Mild	3 (6.4) [5]	1 (2.4) [1]	4 (9.3) [5]	1 (2.5) [2]	9 (5.2) [13]
Moderate	1 (2.1) [1]	0	0	0	1 (0.6) [1]
Severe	0	0	0	0	0
**Subjects reporting AEs with Action Taken**					
None	3 (6.4) [6]	1 (2.4) [1]	4 (9.3) [5]	1 (2.5) [2]	9 (5.2) [14]

Category, n† (%)* [n]#	Treatment Group<br>Lactitol 10 g + Placebo (N=47)	Psyllium 3.5 g + Placebo (N=42)	Lactitol 10 g + Psyllium 3.5 g (N=43)	Placebo (N=40)	Overall (N=172)
Drug Interrupted	0	0	0	0	0
Drug Stopped permanently	0	0	0	0	0
Withdrawal from study	0	0	0	0	0
Unknown	0	0	0	0	0
Other	0	0	0	0	0
**Subjects reporting AEs with relationship**					
Probable	0	0	0	0	0
Possible	0	0	1 (2.3) [1]	0	1 (0.6) [1]
Not Related	3 (6.4) [6]	1 (2.4) [1]	4 (9.3) [4]	1 (2.5) [2]	9 (5.2) [13]
Unlikely	0	0	0	0	0
Not-Classifiable	0	0	0	0	0
**Another treatment given due to AE**					
Yes	1 (2.1) [1]	1 (2.4) [1]	4 (9.3) [5]	1 (2.5) [2]	7 (4.1) [9]
No	2 (4.3) [5]	0	0	0	2 (1.2) [5]
**Subjects reporting AEs by outcome**					
Recovered	3 (6.4) [6]	0	2 (4.7) [2]	0	5 (2.9) [8]
Recovering	0	1 (2.4) [1]	0	1 (2.5) [2]	2 (1.2) [3]
Recovered with sequelae	0	0	0	0	0
Not Recovered	0	0	2 (4.7) [3]	0	2 (1.2) [3]
Fatal	0	0	0	0	0
Unknown	0	0	0	0	0

Category, n† (%)* [n]#	Treatment Group<br>Lactitol 10 g + Placebo (N=47)	Psyllium 3.5 g + Placebo (N=42)	Lactitol 10 g + Psyllium 3.5 g (N=43)	Placebo (N=40)	Overall (N=172)

Note:

† and N: Number of subjects.

* Percentage were calculated by taking the respective column header group count as denominator.

# Number of cases.

Adverse events were coded using MedDRA version 19.1 or later.

Subjects may have reported more than one event per system organ class or preferred term.

Zero frequencies are denoted by "0 (0.0 %).

Abbreviations: adverse events (AEs), treatment-emergent adverse events (TEAEs).

Table S7. Summary of Treatment Emergent Adverse Events- Overall summary- Safety Population(N=172)

	Treatment Group				
**Category, n† (%)* [n]#**	**Lactitol 10 g + Placebo (N=47)**	**Psyllium 3.5 g + Placebo (N=42)**	**Lactitol 10 g + Psyllium 3.5 g (N=43)**	**Placebo (N=40)**	**Overall (N=172)**
**Total number of TEAEs reported**	4	0	4	0	8
**Total number of Non-TEAEs reported**	2	1	1	2	6
**Subjects reporting any TEAEs**	2 (4.3) [4]	0	3 (7.0) [4]	0	5 (2.9) [8]
Subjects reporting 1 TEAE	1 (2.1) [1]	0	2 (4.7) [2]	0	3 (1.7) [3]
Subjects reporting >1 TEAE	1 (2.1) [3]	0	1 (2.3) [2]	0	2 (1.2) [5]
**Subjects reporting TEAEs by seriousness**					
Yes	0	0	0	0	0
No	2 (4.3) [4]	0	3 (7.0) [4]	0	5 (2.9) [8]
**Subjects reporting TEAEs with severity**					
Mild	2 (4.3) [3]	0	3 (7.0) [4]	0	5 (2.9) [7]
Moderate	1 (2.1) [1]	0	0	0	1 (0.6) [1]
Severe	0	0	0	0	0
**Subjects reporting TEAEs with Action Taken**					
None	2 (4.3) [4]	0	3 (7.0) [4]	0	5 (2.9) [8]
Drug Interrupted	0	0	0	0	0
Drug Stopped permanently	0	0	0	0	0
Withdrawal from study	0	0	0	0	0
Unknown	0	0	0	0	0
Other	0	0	0	0	0
**Subjects reporting TEAEs with relationship**					
Probable	0	0	0	0	0
Possible	0	0	1 (2.3) [1]	0	1 (0.6) [1]
Not Related	2 (4.3) [4]	0	3 (7.0) [3]	0	5 (2.9) [7]
Unlikely	0	0	0	0	0
Not-Classifiable	0	0	0	0	0
**Another treatment given due to TEAE**					
Yes	1 (2.1) [1]	0	3 (7.0) [4]	0	4 (2.3) [5]
No	1 (2.1) [3]	0	0	0	1 (0.6) [3]

Category, n† (%)* [n]#	Treatment Group				
	Lactitol 10 g + Placebo (N=47)	Psyllium 3.5 g + Placebo (N=42)	Lactitol 10 g + Psyllium 3.5 g (N=43)	Placebo (N=40)	Overall (N=172)
**Subjects reporting TEAEs by outcome**					
Recovered	2 (4.3) [4]	0	1 (2.3) [1]	0	3 (1.7) [5]
Recovering	0	0	0	0	0
Recovered with sequelae	0	0	0	0	0
Not Recovered	0	0	2 (4.7) [3]	0	2 (1.2) [3]
Fatal	0	0	0	0	0
Unknown	0	0	0	0	0

Note:

† and N: Number of subjects.

* Percentage were calculated by taking the respective column header group count as denominator.

# Number of cases.

Treatment Emergent Adverse events were coded using MedDRA version 19.1 or later.

Subjects may have reported more than one event per system organ class or preferred term.

Zero frequencies were presented by "0 (0.0 %) ".

Abbreviations: treatment-emergent adverse events (TEAEs).
